# Relationships between self-rated oral health, subjective symptoms, oral health behavior and clinical conditions in Japanese university students: a cross-sectional survey at Okayama University

**DOI:** 10.1186/1472-6831-13-62

**Published:** 2013-11-06

**Authors:** Azusa Kojima, Daisuke Ekuni, Shinsuke Mizutani, Michiko Furuta, Koichiro Irie, Tetsuji Azuma, Takaaki Tomofuji, Yoshiaki Iwasaki, Manabu Morita

**Affiliations:** 1 Department of Preventive Dentistry, Okayama University Graduate School of Medicine, Dentistry and Pharmaceutical Sciences, Okayama, Japan; 2 Section of Preventive and Public Health Dentistry, Division of Oral Health, Growth and Development, Faculty of Dental Science Kyushu University, Fukuoka, Japan; 3 Health Service Center, Okayama University, Okayama, Japan

**Keywords:** Young adults, Self-rated oral health, Malocclusion, Temporomandibular disorders, Stomatitis, Behavioral sciences

## Abstract

**Background:**

Self-rated oral health is a valid and useful summary indicator of overall oral health status and quality of life. However, few studies on perception of oral health have been conducted among Japanese young adults. This study investigated whether oral health behavior, subjective oral symptoms, or clinical oral status were associated with self-rated oral health in Japanese young adults.

**Methods:**

This cross-sectional survey included 2,087 students (1,183 males, 904 females), aged 18 and 19 years, at Okayama University, Japan. A self-administered questionnaire was distributed and an oral examination was performed.

**Results:**

In a structural equation modeling analysis, the score of decayed, missing and filled teeth (DMFT) significantly affected self-rated oral health (*p* <0.05) and the effect size was highest. Malocclusion, subjective symptoms of temporomandibular disorders (TMD) and stomatitis, and poor oral health behavior significantly induced self-rated poor oral health with small effect sizes (*p* <0.05). Clinical periodontal conditions and Oral Hygiene Index-simplified were not related to self-rated oral health.

**Conclusion:**

Self-rated oral health was influenced by subjective symptoms of TMD and stomatitis, oral health behavior, the score of DMFT, and malocclusion. The evaluation of these parameters may be a useful approach in routine dental examination to improve self-rated oral health in university students.

## Background

Self-rated health and oral health are key factors that have an impact on well-being and quality of life [1-3]. Self-rated oral health is assessed frequently in epidemiologic studies, including national health surveys, and is a valid and useful summary indicator of overall oral health status [3,4]. There are several reasons for evaluating self-rated oral health: 1) self-reporting helps in the routine diagnostic procedures of dental clinicians [5], 2) assessment of treatment needs requires information about perceived needs [6], 3) assessment of self-rated oral health is relatively simple and may be an easier and complementary method to collect dental information on adolescents and adults [7], and 4) it can be a useful tool for planning and monitoring health services and health promotion interventions [3-8]. Recently, it was suggested that monitoring self-rated oral health in the general population may be a useful way to help people recognize the importance of regular dental checkups [3], and surveillance of oral health in young people should include information on self-rated oral health [9].

Self-rated oral health is associated with clinical and subjective oral factors as well as social factors (socioeconomic status and social capital) [3-5,10,11]. Clinical factors include dental decay, missing teeth, and bleeding gums, whereas subjective factors include reported general health and presence of oral pain [5]. These factors are thought to influence self-rated oral health. Furthermore, self-rated oral health has an impact on well-being and quality of life [1-3], and investigating related factors is important for good self-rated oral health. For example, people who have difficulty eating [3], bad tooth conditions (tooth loss, untreated dental caries, etc.) [11,12], and high scores for decayed, missing, and filled teeth (DMFT) [13] had poor self-rated oral health. However, most studies on perception of oral health in Japan have been conducted in elderly [11] and middle-aged populations [3]. Although information on a special subgroup of young people such as patients or military personnel [13,14] is available, there are few studies targeting general young adults. Because factors associated with self-rated oral health differ by ethnicity [15], it is valuable to identify the determinants of self-rated oral health in Japanese young people.

In addition, university students are in a dynamic transition period of growth and development that bridges adolescence (high school students) and adulthood (people in the community) [16]. At this stage, many of them live away from home for the first time in their life and are faced with the responsibility for their personal health, lifestyle, and behavior. During this period, poor health behavior may affect self-rated oral health. Thus it is important to focus on any factors related to self-rated oral health in university students. We hypothesized that factors such as oral health behavior and clinical oral health status may influence self-rated oral health in Japanese young adults. The purpose of this study was to investigate whether oral health behavior, subjective oral symptoms, or clinical oral status were associated with self-rated oral health in Okayama University students in Japan.

## Methods

### Study population

The setting of our study was Okayama, a mid-sized city in Japan (population 700,000) in the mid-west of Japan. There were 93,699 people aged 15–19 year-old in Okayama in 2011. The ratio of students who went on to universities was 53.9% in Japan, and most first year students are 18 and 19 years old [17]. Okayama University was established in 1949 and is now one of the biggest national universities in Japan. Of 2,441 first year students at Okayama University, a total of 2,395 first-year students underwent a general health examination at the Health Service Center of Okayama University in April 2011. The general health examination is mandatory for first-year students in all departments in the University (medicine, dentistry, pharmacology, science, engineering, literature, economics, law, agriculture and education). Of these participants, 2,319 (96.8%) students volunteered to undergo an oral examination and answer the questionnaire described below. We excluded participants who were ≥20 years old, because most of the participants were 18 or 19 years old and we wanted to avoid age-related factors [18]. The study was approved by the Ethics Committee of Okayama University Graduate School of Medicine, Dentistry and Pharmaceutical Sciences (No. 269). Verbal consent was obtained from all participants.

### Questionnaire

A questionnaire was mailed to participants before the health examination. In addition to age, sex, and general condition, the questionnaire included the following items: perceived oral health, oral health behavior, and subjective oral symptoms.

Self-rated oral health was assessed by the question: 'In general, how do you consider your oral health?’ [4]. The response options were very good, good, fair, poor, and very poor.

To assess oral health behavior, participants were asked to state their daily frequency of toothbrushing by choosing from a list of options (1, 2, or ≥3 times). Participants were also ask to respond yes or no regarding whether they had experienced topical application of fluoride and a regular dental check-up during the past year [18].

In terms of subjective oral symptoms, response to having recurrent aphthous stomatitis was coded as yes or no. Questions to identify temporomandibular disorder (TMD) symptoms included the following: During the past year, 1) have you ever noticed any sounds around your ears? (clicking), 2) have you ever felt pain around the temporomandibular joint (TMJ) while opening your mouth or chewing food? (pain in TMJ), and 3) have you ever had difficulty in opening your mouth? (difficulty in mouth opening) [19]. Each question was answered by selecting a description of awareness (frequently, sometimes, rarely or never) [19].

### Oral examination

One of four dentists (SM, DE, KI, or TA) recorded the oral health status of participants. The DMFT score was used to evaluate dental caries status based on the World Health Organization caries diagnostic criteria [20]. Periodontal condition was assessed using the Community Periodontal Index (CPI) [20]. Ten teeth were selected for periodontal examination: two molars in each posterior sextant and the upper right and lower left central incisors. Measurements were made using a CPI probe (YDM, Tokyo, Japan) at six sites (mesio-buccal, mid-buccal, disto-buccal, disto-lingual, mid-lingual, and mesio-lingual) per tooth. The percentage of teeth exhibiting bleeding on probing (%BOP) was calculated [21]. BOP is an earlier and more sensitive indicator of inflammation than probing pocket depth or visual signs of inflammation (redness and swelling). Thus, we assessed %BOP as an indicator of periodontal disease or gingivitis in this study [21]. The level of dental plaque and calculus was assessed using the Oral Hygiene Index-simplified (OHI-S) [21]. The index has two components: Debris index-simplified and Calculus index-simplified. The degree of debris and calculus deposition was graded on a numeric scale from 0 to 3, divided by the number of sites recorded. The areas examined were the buccal of upper first molar, upper right incisor, and left incisor, and the lingual of lower first molar. Intra- and inter-examiner agreement for the oral examination (score of DMFT and probing pocket depth) was good, as indicated by kappa statistics of more than 0.8.

For malocclusion, a modified version of the Index of Orthodontic Treatment Need (IOTN) was used for each participant. A previous study suggested that the modified IOTN is useful for screening malocclusion by non-specialists in oral health surveys [22]. The dental health component of the modified IOTN consists of a two-grade scale (0 = no definite need for orthodontic treatment and 1 = definite need for orthodontic treatment) with no subcategories. The four dentists, who are not orthodontists, assessed the modified IOTN. In a preliminary check, the kappa value was more than 0.80.

### Statistical analyses

The chi-square test and t-test were used to compare differences between genders for all findings. A *p*<0.05 was considered statistically significant. A statistical program (SPSS version 17.0; IBM, Tokyo, Japan) was used for data analyses. The structural equation modeling (SEM) analysis is used to test the relationship between measured and latent variables. Many researchers have preferred to use the logistic regression or multiple linear regression analysis in a multivariate analysis. These analyses are set on only one dependent variable and enable one to examine direct effects from independent variables to dependent variable, but not indirect effects. In addition, they cannot reveal complex and diverse relationships between independent variables and dependent variables. On the other hand, SEM enables variables to act both as independent and dependent, and has some advantages over multiple-regression techniques for analyzing complex relationships within a conceptual model by allowing the inclusion of latent variables. Latent variables are those that cannot be measured directly but are estimated from measured variables in the model. Relationships between the constructs were assessed using Mplus version 6 (Muthén & Muthén, Los Angeles, CA, USA). The path analysis was performed using weighted least-squares parameter estimates (WLSMV). WLSMV uses a diagonal weight matrix with robust standard errors and mean- and variance-adjusted chi-square test statistics. For the global fit indices, a non-significant chi-square indicates that the data do not significantly differ from the hypotheses represented by the model; for comparative fit index (CFI) and Tucker-Lewis index (TLI), fit indices of above 0.90 (preferably above 0.95) indicate a well-fitting model [21]. For root mean square error of approximation (RMSEA), a fit of less than 0.05 indicates a well-fitting model [21]. We used a significance level of *p*<0.05 for the regression coefficients. We set “TMD”, “Oral health behavior,” and “Clinical periodontal conditions” as latent variables. The latent construct “TMD” comprised “Pain in TMJ,” “Clicking,” and “Difficulty in mouth opening”; “Oral health behavior” comprised “Frequency of toothbrushing,” “Regular check-up,” and “Application of fluoride”; and “Clinical periodontal conditions” comprised “CPI” and “%BOP”. Low scores indicated a good situation, that is, for self-rated oral health 1 = very good, 2 = good, 3 = fair, 4 = poor, and 5 = very poor; for TMD, 1 = never, 2 = rarely, 3 = sometimes, and 4 = frequently; and for toothbrushing frequency, 1 = three times daily or more, 2 = two times, and 3 = 1 time or less. For other parameters, 1 = yes and 2 = no.

The Cohen’s effect size was assessed using correlation coefficients or standardized coefficient corresponded to r; the small, medium, and large effect sizes were 0.10, 0.30, and 0.50, respectively [21].

## Results

We excluded 120 participants who were ≥20 years old and 112 participants who had provided incomplete data in their questionnaires. As a result, data from 2,087 students (1,183 males, 904 females) aged 18 and 19 years were analyzed. Table 1 shows the data of questionnaires. Overall, 541 (25.9%) participants had poor self-rated oral health. There were no significant differences in self-rated oral health between males and females. The percentage of participants with subjective symptom of stomatitis was 25.1%. The percentages of participants who had never experienced clicking in TMJ, pain in TMJ, and difficulty in mouth opening were 57.3%, 79.9%, and 80.5%, respectively. There was a significant difference in the percentage of participants who had difficulty in mouth opening between males and females (*p*<0.05).

**Table 1:** **Data of questionnaires**: **latent and measured variables** (**N** = **2**,**087**)

**Variable**		**Males**	**Females**	***p-*** **value**^**†**^
		**(n = 1,183)**	**(n = 904)**	
Self-rated oral health	Very good	53 (4.5)*	45 (5.0)	0.565
	Good	204 (17.2)	169 (18.7)	
	Fair	605 (51.1)	470 (52.0)	
	Poor	275 (23.2)	193 (21.3)	
	Very poor	46 (3.9)	27 (3.0)	
Oral health behavior				
Daily frequency of toothbrushing	1 time	309 (26.1)	85 (9.4)	<0.001
	2 times	797 (67.4)	693 (76.7)	
	3 times or more	77 (6.5)	126 (13.9)	
Experience of topical application of fluoride	Yes	346 (29.2)	365 (40.4)	<0.001
	No	837 (80.8)	539 (59.6)	
Regular check-up	Yes	138 (11.7)	159 (17.6)	<0.001
	No	1045 (88.3)	745 (82.4)	
Symptom of recurrent aphthous stomatitis	Yes	281 (23.8)	243 (26.9)	0.103
	No	902 (76.2)	661 (73.1)	
Temporomandibular disorders				
Clicking	Frequently	135 (11.4)	111 (12.3)	0.172
	Sometimes	143 (12.1)	137 (15.2)	
	Rarely	211 (17.8)	155 (17.1)	
	Never	694 (58.7)	501 (55.4)	
Pain in temporomandibular joint	Frequently	15 (1.3)	18 (2.0)	0.066
	Sometimes	54 (4.6)	62 (6.9)	
	Rarely	158 (13.4)	112 (12.4)	
	Never	956 (80.8)	712 (78.8)	
Difficulty in mouth opening	Frequently	30 (2.5)	26 (2.9)	0.016
	Sometimes	71 (6.0)	71 (7.9)	
	Rarely	101 (8.5)	107 (11.8)	
	Never	981 (82.9)	700 (77.4)	

*number (%).

^†^Chi-square test.

Objective data are shown in Table 2. There were significant differences between males and females in all variables except for the CPI score.

**Table 2:** **Clinical parameters**: **latent and measured variables** (**N** = **2**,**087**)

**Variable**		**Males (n = 1, 183)**	**Females (n = 904)**	***p-*** **value**
Decayed, missing, and filled teeth score		2.1 ± 2.8*	2.6 ± 3.1	0.001^†^
Oral hygiene index-simplified		0.71 ± 0.56	0.57 ± 0.47	<0.001^†^
Calculus index-simplified		0.18 ± 0.24	0.15 ± 0.21	0.002^†^
Debris index-simplified		0.52 ± 0.40	0.42 ± 0.35	<0.001^†^
Malocclusion	+	297 (25.1)^‡^	277 (30.6)	0.005^§^
	-	886 (74.9)	627 (69.4)	
Clinical periodontal conditions				
Community Periodontal Index	0	170 (14.4)	137 (15.2)	0.211^§^
	1	221 (18.7)	179 (19.8)	
	2	677 (57.2)	476 (62.7)	
	3	113 (9.6)	110 (12.2)	
	4	2 (0.2)	2 (0.2)	
Bleeding on probing (%)		30.2 ± 26.3	27.8 ± 26.2	0.046^†^

*Mean ± SD (%).

^†^t-test.

^‡^number (%).

^§^Chi-square test.

**Figure 1:**
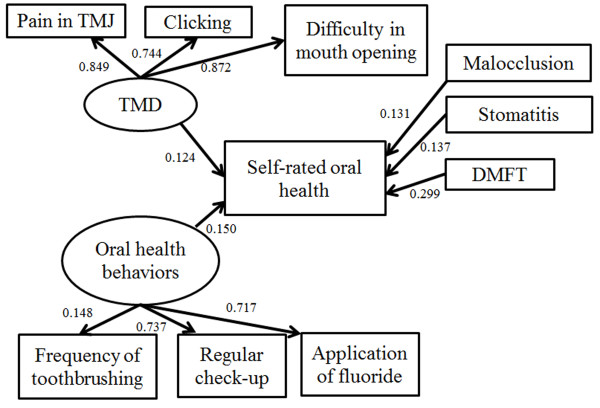
**The final structural model.** Rectangles indicate observed variables and ovals show latent variables. The values of single-headed arrows indicate the standardized coefficient. All pathways are significant (*p*<0.05). Self-rated oral health was associated with subjective symptoms of TMD and stomatitis, oral health behavior, the DMFT score, and malocclusion. TMD, temporomandibular disorders; TMJ, temporomandibular joint; DMFT, decayed, missing, and filled teeth.

We estimated a final model with all hypothesized pathways using SEM analysis. The value of chi-square was significant because of our large sample size (×^2^=77.99, df=31, and *p*<0.0001). CFI, TLI, and RMSEA values indicated good model-data fit (0.986, 0.980, and 0.027, respectively). Figure 1 shows the parameters estimated for the final structural model. The model showed that (i) the score of DMFT affected self-rated oral health and the effect size was highest; (ii) malocclusion affected self-rated oral health with a small effect size; (iii) subjective symptoms of TMD and stomatitis affected self-rated oral health with a small effect size; and (iv) poor oral health behavior induced self-rated poor oral health with a small effect size. All pathways were significant (*p*<0.05). Clinical periodontal conditions and OHI-S, as well as sex, were excluded from the final model, as good model-data fit was not obtained when these conditions were included.

## Discussion

Self-rated oral health is a valid and useful summary indicator of overall oral health status used in epidemiologic studies [3,4]. The main factors associated with self-rated oral health include both clinical and subjective oral factors [5]. However, there is little information about whether malocclusion (a clinical factor) and symptoms of TMD and stomatitis (subjective factors) affect self-rated oral health. To investigate these factors is important for good self-rated oral health and quality of life. Thus, this study focused on showing the relationship between self-rated oral health and these factors in Japanese young adults.

In this study, self-rated oral health was related to malocclusion, subjective symptoms of TMD, and stomatitis as well as the score of DMFT and oral health behavior in first-year university students. Self-rated oral health is a one of the key factors that has an impact on well-being and quality of life [1-3]. Although the effect sizes of the paths from malocclusion, subjective symptoms of TMD, and stomatitis to self-rated oral health were small, prevention/management of these oral diseases might contribute to achieving better quality of life in the young adult population.

The presence of malocclusion affected self-rated poor oral health in this study. Malocclusion has physical, psychological, and social effects on quality of life as well as oral conditions [23]. Negative impacts on daily performance attributed to malocclusion may contribute to psychological stress in young Japanese adults [22]. Thus psychological stress by malocclusion may relate to self-rated poor oral health.

A very good reliability and high validity has been observed for self-reported TMJ pain questions, and these questions have been used to screen for TMJ pain in the adolescent population [24]. Another study also reported good reproducibility and validity for self-reported TMJ conditions (clicking, pain in TMJ, and difficulty in mouth opening) [25], which was similar to our questionnaire. Using such questionnaires, a significant relationship between subjective TMD symptoms and self-rated oral health was observed. Appearance of oral pain affects self-rated oral health [5]. Oral health-related quality of life is negatively affected among TMD patients [26]. Therefore, the self-reported questions for TMJ could detect not only temporomandibular disorders, but also self-rated poor oral health.

Oral mucosal diseases are common, and many of them are of unknown cause [27]. One of the oral mucosal diseases, recurrent aphthous stomatitis, affects about 0.5-60% of the population [28,29]. Recurrent aphthous stomatitis affects quality of life [30]. Because oral mucosal diseases can seriously impair quality of life and affect various aspects of life [27], recurrent aphthous stomatitis may influence self-rated oral health, which relates to quality of life. In this study, subjective symptoms of recurrent aphthous stomatitis were associated with self-rated poor oral health. The results support our hypothesis.

The scores of DMFT and oral health behavior were related to self-rated oral health, that is, participants with a high DMFT score and poor oral health behavior believed that they had poor oral health. These results were similar to those of other studies [3-13]. Furthermore, the DMFT score and oral health behavior had a higher association with self-rated oral health compared with other parameters (malocclusion and symptoms of TMD and stomatitis) in this study. The effect size of the path from the DMFT score was medium and highest in all parameters. The effect size of oral health behavior ranked second, although the size was small. Therefore, these two parameters can be most effective in changing self-rated perceived oral health. The mechanisms are not clear but there may be potential interactions. First, the DMFT score had a higher association with self-rated oral health in this study. A high DMFT score is correlated with anxiety [13]. Anxiety affects self-rated oral health [4]. Thus, anxiety associated with caries experience might strongly influence self-rated oral health. Furthermore, significant correlations were observed between self-rated oral health and number of decayed, missing or filled teeth using the Spearman’s correlation coefficient (r = 0.138, 0.068, 0.248, respectively) (*p*<0.01). The findings also supported the relationship between self-rated oral health and caries experience. On the other hand, regular dental check-up had the highest contribution to oral health behavior in this study. Regular dental check-up directly affects self-rated oral health [31], which may contribute to higher association between oral health behavior and self-rated oral health. However, further studies are required to investigate the details.

Clinical periodontal conditions such as CPI score and %BOP as well as OHI-S were excluded from the final model and did not show a significant effect on self-rated oral health in this study. The reason may be because young students with periodontal diseases have minimal symptoms. This finding was similar to reports in other countries [14] or other populations (middle-aged adults) in Japan [3]. People who cannot recognize and acknowledge their deteriorating periodontal condition accurately may be at increased risk of delaying access to dental care [3]. Thus, prevention of periodontal disease in younger populations may require earlier recognition of the signs and symptoms of these diseases as well as clinical examination.

In Japan, health examinations are implemented on a regular basis according to the school health and safety law. Self-rated oral health is one of the key factors that have an impact on well-being and quality of life [1-3]. Because preventing impairment of quality of life at an early stage by controlling associated factors in younger populations is important, self-rated oral health should be monitored by regular health examination. Further studies will be required to investigate whether improvement of factors related to self-rated oral health contributes to self-rated good oral health and well-being. From this viewpoint, the routine oral examination should include investigating subjective symptoms of TMD and stomatitis, as well as malocclusion.

SEM analysis was used in this study to explore the complex relationship between self-rated oral health and possible related factors. In a multivariate analysis, many researchers prefer to use the logistic regression or multiple linear regression analysis. These analyses are set on only one dependent variable and cannot reveal complex and diverse relationships between independent variables and dependent variables. On the other hand, the SEM analysis procedures can examine both direct and indirect effects of variables, as well as explore the complex causal relationship involved in the process [3,18].

Our study had several limitations. First, this was a cross-sectional study. A prospective cohort or intervention studies may provide information beyond what we present here. Second, all participants were recruited from among students at Okayama University. This may limit the ability to extrapolate these findings to the general population. Third, we did not consider possible related factors, such as socioeconomic status, [5] social capital, [4] and psychosocial valuables. Fourth, we did not investigate the existence of subjective oral symptoms, such as pain due to dental caries. However, in this study, most lesions identified were early carious lesions. As a result, few students actually consulted the dentist about the pain due to dental caries at the oral examination setting, which suggests that our results may be relatively unaffected by this limitation. Furthermore, the data obtained were limited because our study was combined with a routine health screening.

## Conclusions

Self-rated oral health was associated with subjective symptoms of TMD and stomatitis, oral health behaviors, the DMFT score, and malocclusion in first-year students at Okayama University. The effect size of the path from the DMFT score to self-rated oral health was highest for all parameters, and the effect size of oral health behavior ranked second. The evaluation of TMD, stomatitis, and malocclusion as well as the DMFT score and oral health behavior may be a useful approach in routine dental examinations to improve self-rated oral health and quality of life in university students.

## Abbreviations

DMFT: Decayed, missing, and filled teeth; TMD: Temporomandibular disorders; TMJ: Temporomandibular joint; CPI: Community periodontal index; BOP: Bleeding on probing; OHI-S: Oral Hygiene index-simplified; IOTN: Index of orthodontic treatment need; SEM: Structural equation modeling; WLSMV: Weighted least-squares parameter estimates; CFI: comparative fit index; TLI: Tucker-Lewis index; RMSEA: Root mean square error of approximation

## Competing interests

The authors declare that they have no competing interests.

## Authors’ contributions

AK, DE, SM, MF, KI, TA, TT, YI, and MM conceived and planned the project. AK, DE, SM, TT, and MM wrote the manuscript. DE, MF, KI, and TA conducted oral examinations. YI conducted general examinations. AK, DE, SM, MF, KI, TA, and TT performed data entry. AK, SM, and DE conducted the statistical analysis. All authors read and approved the final manuscript.

## Pre-publication history

The pre-publication history for this paper can be accessed here:

http://www.biomedcentral.com/1472-6831/13/62/prepub
